# Particles induce apical plasma membrane enlargement in epithelial lung cell line depending on particle surface area dose

**DOI:** 10.1186/1465-9921-10-22

**Published:** 2009-03-12

**Authors:** Christina Brandenberger, Barbara Rothen-Rutishauser, Fabian Blank, Peter Gehr, Christian Mühlfeld

**Affiliations:** 1 Institute of Anatomy, University of Bern, Baltzerstrasse 2, CH-3000 Bern 9, Switzerland; 2 Telethon Institute for Child Health Research, 100 Roberts Road, Subiaco, Perth, WA 6008, Australia; 3 Institute of Anatomy and Cell Biology, Justus-Liebig-University Giessen, Aulweg 123, D-35385 Giessen, Germany

## Abstract

**Background:**

Airborne particles entering the respiratory tract may interact with the apical plasma membrane (APM) of epithelial cells and enter them. Differences in the entering mechanisms of fine (between 0.1 μm and 2.5 μm) and ultrafine ( ≤ 0.1 μm) particles may be associated with different effects on the APM. Therefore, we studied particle-induced changes in APM surface area in relation to applied and intracellular particle size, surface and number.

**Methods:**

Human pulmonary epithelial cells (A549 cell line) were incubated with various concentrations of different sized fluorescent polystyrene spheres without surface charge (∅ fine – 1.062 μm, ultrafine – 0.041 μm) by submersed exposure for 24 h. APM surface area of A549 cells was estimated by design-based stereology and transmission electron microscopy. Intracellular particles were visualized and quantified by confocal laser scanning microscopy.

**Results:**

Particle exposure induced an increase in APM surface area compared to negative control (p < 0.01) at the same surface area concentration of fine and ultrafine particles a finding not observed at low particle concentrations. Ultrafine particle entering was less pronounced than fine particle entering into epithelial cells, however, at the same particle surface area dose, the number of intracellular ultrafine particles was higher than that of fine particles. The number of intracellular particles showed a stronger increase for fine than for ultrafine particles at rising particle concentrations.

**Conclusion:**

This study demonstrates a particle-induced enlargement of the APM surface area of a pulmonary epithelial cell line, depending on particle surface area dose. Particle uptake by epithelial cells does not seem to be responsible for this effect. We propose that direct interactions between particle surface area and cell membrane cause the enlargement of the APM.

## Background

With every breath, large numbers of airborne particles enter the human body and may encounter the vast epithelial surface of the respiratory tract. In recent years, there has been increasing interest in the interactions between particles and structures of the respiratory tract (for recent reviews see [1-4]), particularly because of a growing body of epidemiological and experimental literature suggesting adverse respiratory and cardiovascular human health effects due to airborne particle exposure [5-10]. Particular focus has been placed on combustion-derived fine (diameter between 2.5 μm and 0.1 μm) and ultrafine (diameter < 0.1 μm) particles, as well as manufactured nanoparticles (at least in one dimension < 0.1 μm) [11].

Inhaled particles that get into contact with surfactant are immediately displaced to the watery hypophase [12,13] where they may interact with hydrophilic proteins [14,15] or cells, such as alveolar macrophages or epithelial cells [16,17]. The interaction of particles with the epithelial cells includes endocytosis, potentially followed by intracellular storage, transcytosis or exocytosis. The mechanism by which particles of different sizes are endocytosed has been subject to thorough investigations and there is convincing evidence that different mechanisms are involved in particle uptake, including phagocytosis, macropinocytosis, clathrin-mediated endocytosis, caveolae/raft-mediated endocytosis and direct entering mechanisms, summarized by the term adhesive interactions [18-23]. However, the equilibrium between endocytosis and exocytosis is highly regulated in intact cells and any interfering process may alter the balance of the apical plasma membrane (APM). Endocytosis and exocytosis involve trafficking of membrane lipids to and from the APM. For example, cell deformation stress induces lipid trafficking in lung epithelial cells, thus increasing apical plasma membrane surface [24]. Inhibition of deformation-induced lipid trafficking leads to an increased probability of cell wounding and a decreased probability of wound resealing [25]. Therefore, lipid trafficking to the APM of pulmonary epithelial cells is thought to be a protective stress response. Mechanisms by which lipid trafficking to the APM occurs may include exosomes [26,27] or enlargosomes [28].

Several studies have shown that particles of various sizes may be internalized or exocytosed by epithelial cells [29-31], however, the effects of particle exposure on the plasma membrane have not been addressed so far. Since this interaction may alter cell metabolism and integrity, it is of importance to understand the changes of the APM of epithelial cells upon particle exposure. We therefore hypothesized that particle exposure leads to a decrease in APM surface area due to particle endocytosis or to an increase in APM surface area due to stress induced exocytosis. To address this question, we exposed an immortalized human pulmonary epithelial cell line (A549) [32] to various concentrations of non-soluble, low-toxicity fluorescent polystyrene particles of 1 μm and 0.05 μm diameter. The different particle concentrations were chosen to analyze which particle characteristic (number, surface or volume/mass) determines the effects of particles on the changes in APM surface area. The latter was quantified by design-based stereology at the electron microscopic level. Additionally, we hypothesized that differences in particle-induced APM surface area are related to the uptake of particles by the A549 cells. Therefore, we quantified the number of intracellular particles by confocal laser scanning microscopy (LSM) followed by application of a deconvolution algorithm [20].

## Methods

### A549 cultures

The A549 cell line was obtained from American Tissue Type Culture Collection (LGC Promochem, Molsheim, France). Cells (passage number 8 to 50) were maintained in RPMI 1640 medium (w/25 mM HEPES, LabForce AG, Nunningen, Switzerland) supplemented with 1% L-Glutamine (LabForce AG), 1% penicillin/streptomycin (Gibco BRL, Life Technologies, Basel, Switzerland), and 10% fetal calf serum (LabForce AG, Nunningen, Switzerland). Cells were seeded at a density of 0.5 × 10^6 ^cells/mL on BD Falcon™ cell culture inserts (High pore density PET membranes for 6-well plates with a growth area of 4.2 cm^2 ^and 3.0 μm pores in diameter; Becton Dickinson, Allschwil, Switzerland). Inserts were placed in BD Falcon™ tissue culture plates with 2 mL medium in the upper and 3 mL in the lower chamber. Medium was changed twice a week. Before particle exposure, cells were grown on inserts submersed in medium for 7 d to grow to confluence. The confluency of the cell layer was confirmed by (LSM) resulting in an average cell density of 6000 ± 400 cells/mm^2^.

### Particles

**Figure 1:**
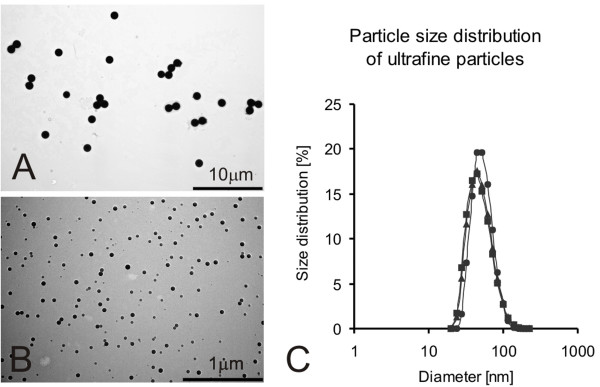
**Particle size characteristics**. Particles were visualized by transmission electron microscopy verifying that no large agglomerates were present (A: 1 μm particles; B: 0.05 μm particles). Ultrafine particle size in RPMI medium was further analyzed by dynamic light scattering. The size distributions from three individual measurements show that the majority of ultrafine particles in RPMI medium are present as single particles or small agglomerates of two to three particles.

Commercially available particles were used: 1 μm and 0.05 μm Fluoresbrite™ plain yellow green polystyrene microspheres (Polysciences, Chemie Brunschwig AG, Basel, Switzerland) with an Excitation/Emission wavelength of 441 nm/486 nm respectively. The particles have no surface charge and are photostable at lysosomal pH. The effective particle diameters are 1.062 μm ± 0.023 μm and 0.041 μm. Standard deviation was not provided for ultrafine particles by the supplier. Estimations from electron microscopic figures and size distribution measurements indicate a standard deviation of approximately 0.015 μm. All calculations and dilutions are based on effective diameters. For better readability the terms of 1 μm and 0.05 μm particles are used throughout the manuscript. Polystyrene particles were diluted in RPMI 1640 medium without serum and adjusted to the desired particle concentration. The agglomeration status of the ultrafine particles was analyzed using a Zetasizer NanoS (Malvern, Herrenberg, Germany) for measurement of particle size distribution in RPMI medium, resulting in a mean distribution of 52.3 nm with a width of 41.9 nm. Furthermore, both particle types were visualized by transmission electron microscopy for verification of particle size (Figure 1). Calculations to determine number, surface area and mass of the particles were performed according to the supplier's manual and applying a model of spherical beads (Table 1). The particle dilutions were sonicated for 5 min prior to incubation with the cells, in order to avoid agglomeration. For exposure, the medium in the cell culture inserts was removed and replaced with 1.5 mL fresh medium in the lower chamber and 333 μL particle suspension in the upper chamber. Cells were incubated with particles for 24 h. Each experiment was repeated 3 to 5 times. The particle concentrations for the different experiments are summarized in Table 1. All particle doses used in this manuscript refer to the exposure of one cell culture transwell (4.2 cm^2^).

**Table 1:** Dose metrics of the different experiments

**Particle size**	**Particle number per well**	**Particle surface area [μm^2 ^per well]**	**Particle volume [μm^3 ^per well]**
APM experiments			

1 μm	3 × 10^7^	1.1 × 10^8^	1.9 × 10^7^

1 μm	6 × 10^8^	2.1 × 10^9^	3.8 × 10^8^

0.05 μm	3 × 10^7^	1.6 × 10^5^	1.1 × 10^3^

0.05 μm	6 × 10^8^	3.2 × 10^6^	2.2 × 10^4^

0.05 μm	4.5 × 10^11^	2.4 × 10^9^	1.6 × 10^7^

Constant particle number or surface area exposure			

1 μm	1 × 10^7^	3.5 × 10^7^	6.3 × 10^6^

1 μm	3 × 10^7^	1.1 × 10^8^	1.9 × 10^7^

0.05 μm	3 × 10^7^	1.6 × 10^5^	1.1 × 10^3^

0.05 μm	6.7 × 10^9^	3.5 × 10^7^	2.4 × 10^6^

Concentration dependent particle entering			

1 μm	1 × 10^7^	3.5 × 10^7^	6.3 × 10^6^

1 μm	3 × 10^7^	1.1 × 10^8^	1.9 × 10^7^

1 μm	6 × 10^7^	2.1 × 10^8^	3.8 × 10^7^

1 μm	9 × 10^7^	3.2 × 10^8^	5.6 × 10^7^

0.05 μm	3 × 10^7^	1.6 × 10^5^	1.1 × 10^3^

0.05 μm	6 × 10^7^	3.2 × 10^5^	2.2 × 10^3^

0.05 μm	6 × 10^8^	3.2 × 10^6^	2.2 × 10^4^

0.05 μm	6 × 10^9^	3.2 × 10^7^	2.2 × 10^5^

Note: Particle dose per cell culture well was based on particle numbers. Corresponding particle surface area was calculated for spherical particles. All calculations are based on the effective diameters of 0.041 μm and 1.062 μm, respectively (see Material and Methods).

### Estimation of apical plasma membrane surface area

To evaluate the effect of 1 μm and 0.05 μm particle exposure on APM surface area of the cells, we estimated the APM surface area per cell using design-based stereology. Cells on insert membrane were fixed with 2.5% glutaraldehyde in 0.03 M potassium phosphate buffer for at least 24 h. Cells were then washed in buffer, post-fixed with 1% osmium tetroxide in sodium cacodylate buffer, washed with maleate, and stained en bloc with 0.5% uranylacetate in maleate buffer. After additional washing, the cells were dehydrated in an ascending ethanol series, and embedded in epon [33]. From the embedded cells, semi- and ultrathin sections were cut parallel to the vertical axis of the cells. These served as vertical sections which allows sound information on the orientation of the cells and predetermines the use of certain stereological techniques, such as cycloid test lines instead of linear test lines [34].

Stereology provides a set of methods which allow the estimation of three-dimensional structural features (number, length, surface area or volume) from two-dimensional sections. All parameters are first determined as densities, i.e. as estimate per unit reference volume, and are then converted to the total value by multiplication with the reference volume. Semithin sections were mounted on glass slides, stained with toluidine blue, sealed with a coverslip and investigated using an Axioskope light microscope equipped with a computer assisted stereology tool (CAST 2.0, Olympus, Ballerup, Denmark), at an objective lens magnification of 40×. For estimation of the mean volume of A549 cells, a number-weighted sampling procedure was used by application of the single section dissector [35,36]. Thus, every time a nucleolus was observed in an A549 nucleus this cell was sampled for cell volume estimation by the vertical rotator [37]. The rotator is a local stereological tool used to estimate the volume of a biological particle from a two-dimensional section. From these results the number-weighted mean volume of A549 cells was estimated for each experiment. Ultrathin sections were mounted on copper grids, stained with lead citrate and uranyl acetate and were investigated with a Philips CM12 transmission electron microscope (FEI Co. Philips Electron Optics, Zürich, Switzerland) at a primary magnification of 4,400×. Test fields showing A549 cells were chosen by systematic uniform random sampling [38], i.e. the first test field was chosen randomly and predetermined the locations of all subsequent test fields. A cycloid test line system [34] was projected onto each test field with the vertical axis of the test system aligned to the vertical axis of the cells. Intersections of the cycloid test lines with the APM were counted. According to S_V_: = 2*I/L_T _the surface density (S_V_) of the APM was calculated from the number of intersections (I) and the total length of the test line (L_T_) hitting the reference space [39]. The total APM surface area per A549 cell was then calculated by multiplying the surface density with the number-weighted mean volume of A549 cells.

### Estimation of the number of intracellular particles

After incubation with particles, the cells kept on membrane were washed in phosphate buffered saline (PBS, 10 mM, pH 7.4: 130 mM NaCl, Na2HPO4, KH2PO4) and fixed for 15 min at room temperature in 3% paraformaldehyde in PBS. Fixed cells were treated with 0.1 M glycine in PBS for 5 min and permeabilized in 0.2% Triton X-100 in PBS for 15 min at room temperature. The cells were incubated with Phalloidin rhodamine (dilution 1:100, R-415, Molecular Probes, Invitrogen AG, Basel, Switzerland) for 60 min at room temperature. Preparations for optical analysis were mounted in PBS:glycerol (2:1) containing 170 mg/mL Mowiol 4–88 (Calbiochem, VWR International AG, Dietikon, Switzerland).

**Figure 2:**
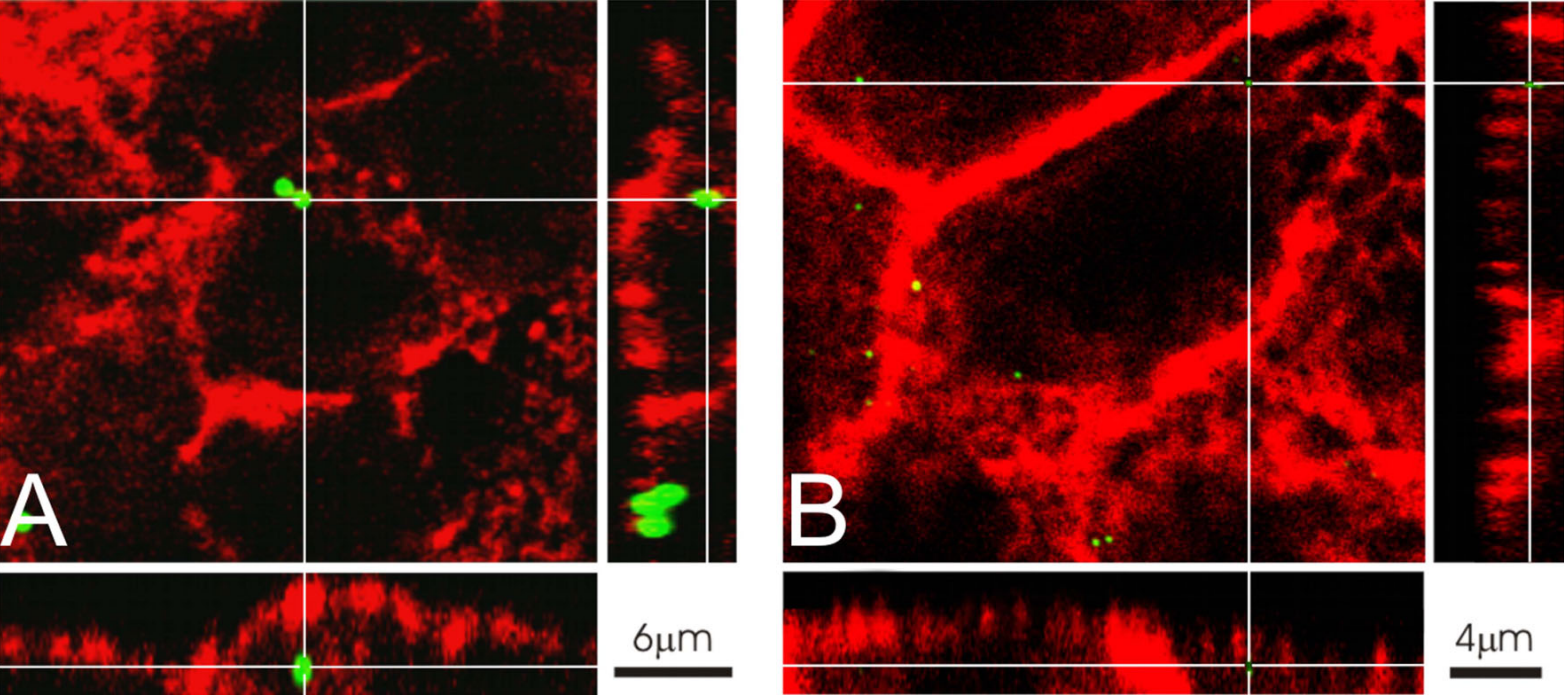
**Visualization of fine and ultrafine particles by confocal laser scanning microscopy**. Figure A illustrates the appearance of 1 μm fluorescent polystyrene particles (green) inside A549 cells. Figure B illustrates the appearance of 0.05 μm fluorescent particles (green) inside A549 cells after application of a deconvolution algorithm. For visualization of the cells, the actin cytoskeleton was stained with phalloidine-rhodamine (red). The panels on the right and at the bottom of each figure show the corresponding y/z and x/z projection, respectively.

A Zeiss LSM 510 Meta with an inverted Zeiss microscope (Axiovert 200 M, Lasers: HeNe 633 nm, HeNe 543 nm, and Ar 488 nm) was used. Image processing and visualization was performed using IMARIS, a 3D multi-channel image processing software for confocal microscopic images (Bitplane AG, Zurich, Switzerland). For the localization and visualization of particles at high resolution a deconvolution algorithm was applied using the Huygens 2 software (Scientific Volume Imaging B. V., Hilversum, Netherlands) in order to increase axial and lateral resolutions and to decrease noise (Figure 2), [40].

After the image acquisition, the total particle number in the scans was counted with the particle tracking software Diacount (Semasopht, Lausanne, Switzerland; ) [20]. For each experimental sample, ten different fields of view were chosen randomly and scanned with LSM. The intracellular number of particles counted in a defined specimen area scanned by LSM was extrapolated to an area of one mm^2^.

### IL8 ELISA

The detection of IL-8 protein released to the culture medium was carried out following the supplier's protocol of the DuoSet ELISA Development Kit (R&D Systems, Catalogue Number: DY 208, Oxon, UK). At 24 h after start of the exposure, 1 mL medium from the lower exposure chamber was sampled and immediately frozen and stored at -70°C until performing the assay. Before use, the samples were thawed from -70°C and centrifuged at 3'000 rpm for 10 min to get rid of particles in the medium. The assay was done in triplicate and the experiments were repeated five times. Samples for IL8 ELISA were diluted in PBS (1:10) and an IL-8 standard range from 2 ng/mL to 0.03 ng/mL was applied. Exposure to TNFα(10 ng/mL) was performed as a positive control for IL-8 induction.

The optical density was detected with an ELISA reader, (BioRad, Hempel Hempstead, UK) at a wavelength of 450 nm. The amount of IL-8 was determined by comparing the absorbance of the samples with standard recombinant human IL-8.

### Cytotoxicity

Cytotoxicity was ascertained by measuring lactate dehydrogenase (LDH) released from necrotic cells. The test was performed with the Cytotoxicity Detection Kit (Roche Applied Science, Mannheim, Germany) according to the supplier's manual. Briefly, 100 μL cell culture medium from the lower chamber and 100 μL freshly prepared colour reagent (Diaphorase/NAD+ mixture with iodotetrazolium chloride/sodium lactate) were mixed and incubated for 20 min. Colour reaction was measured immediately after incubation at wave length 490 nm with an ELISA reader (Bio Rad, Hempel Hempstead, UK). The samples were measured in triplicates and experiments were repeated five times.

The percentage of cytotoxicity was calculated from a positive control with lysed cells (100% cytotoxicity). Cell lysis was performed with 2% Triton-X solution in cell culture medium for 30 min.

### Transcription of key genes required for lipid synthesis and uptake

RNA isolation was done with the Qiagen RNeasy Mini Kit (Qiagen AG, Basel, Switzerland). The cells were released from the cell culture membrane with a cell scratcher and the provided lysis buffer. The cell lysate was then centrifuged in shredder columns (QIAshredder, Qiagen AG, Basel, Switzerland) for 2 min at 13'000 rpm. The isolation was performed according to the supplier's manual including a step of DNA digestion (Qiagen AG, Basel, Switzerland). The purified RNA was eluted in 30 μL pure H_2_O and stored at -70°C.

The RNA concentration was measured with the Nano-Drop-Photometer (NanoDrop ND100 PeqLab, Germany). Transcription was performed with a total amount 0.5 μg RNA in a volume of 20 μL reaction mixture with the Omniskript kit (Qiagen AG, Basel, Switzerland). CDNA was diluted to a concentration of 66 ng/μl and stored at -20°C. The reaction mixture for quantitative real-time PRC contained 160 ng cDNA, SYBER Green Jump Start (Sigma-Aldrich, Buchs Switzerland) and 0.4 μM forward and reverses primer. Primer sequences were obtained from Castoreno et al. 2005 [41]. The thermo cyclic reaction and software analysis was performed with the 7900 HT Fast Real-Time PCR System (Applied Biosystem, Rotkreuz, Switzerland). Experiments were repeated three times at all exposure times and concentrations.

### Statistics

The statistical analyses were carried out with the commercial statistical package SigmaSTAT 3.5 (Systat Software Inc., Erkrath, Germany). Due to the small sample sizes, nonparametric tests were used. Kruskal-Wallis One Way Analysis of variance (ANOVA) on Ranks was performed if more than two groups were compared. If p < 0.05, multiple comparisons were performed using Dunn's method. For comparison of two groups, Mann-Whitney u test was used. Differences were considered significant at p < 0.05.

## Results

### Quantification of total apical cell membrane surface

Before quantification of the APM, we studied the ultrastructure of epithelial cells exposed to different concentrations of 1 μm and 0.05 μm particles qualitatively. At a concentration of 6 × 10^9 ^of 1 μm particles per cell culture well (4.2 cm^2^), most of the cells were apoptotic or necrotic as seen in transmission electron micrographs. Therefore, the highest concentration of 1 μm particles for cell membrane investigations was set at 6 × 10^8 ^particles per well. The concentrations of 0.05 μm particles were chosen to relate particle number and surface area to the observed effects on APM after exposure to 1 μm particles (Table 1).

**Figure 3:**
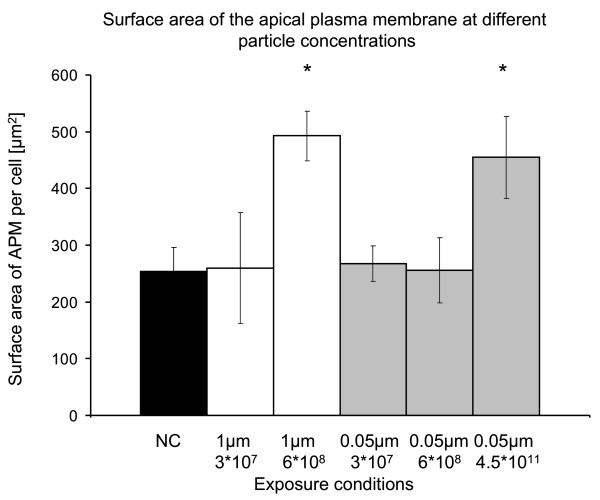
**Surface area of the apical plasma membrane of A549 cells at different particle concentrations**. * = p < 0.01 vs. negative control (NC). The mass of 3 × 10^7 ^1 μm particles and the surface area of 6 × 10^8 ^1 μm particles are approximately equal to the mass and surface area of 4.5 × 10^11 ^0.05 μm particles, respectively. Increases in the surface area of the APM were observed at the same particle surface area concentration exposed to the cells. n = 5.

**Figure 4:**
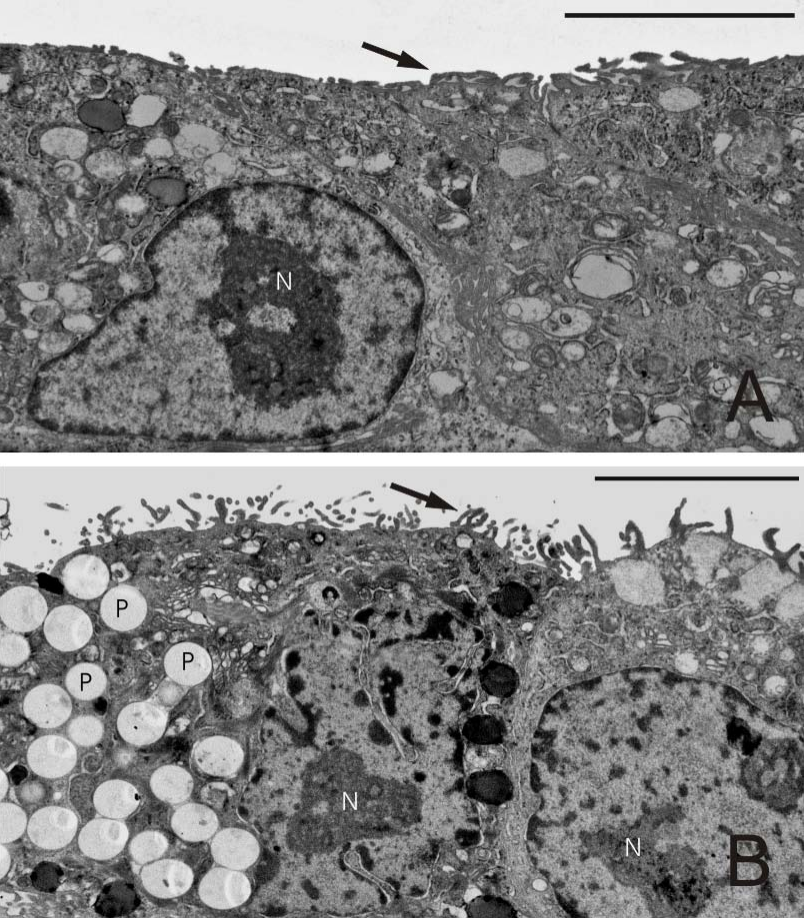
**Electron micrographs of the apical plasma membrane of A549 cells**. A: Control experiments without particle exposure. B: Exposure to 6 × 10^8 ^1 μm particles. Note the changes in APM in comparison with A. Numerous particles (P) taken up by the epithelial cells are found inside the cells. N = Nucleus. Scale bar = 5 μm.

Table 2 summarizes the stereological data and Figure 3 visualizes the results of the mean APM surface area per cell measured by design-based stereology. Upon exposure to 3 × 10^7 ^fine or ultrafine particles, there were no changes in APM surface area. At 6 × 10^8 ^1 μm particles a significant increase in plasma membrane surface was observed which was already evident qualitatively. The increase in apical plasma membrane was reflected by microvilli-like cellular surface extensions (Figure 4). Upon exposure to the same number of 0.05 μm particles (6 × 10^8^), the surface area of the APM remained at control levels, indicating that particle number does not correlate with particle-induced APM surface area changes. However, when the cells were exposed to the corresponding particle surface area concentration (4.5 × 10^11 ^0.05 μm particles per well) a significant increase in APM surface area was observed, which did not differ from that induced by 1 μm particles at 6 × 10^8 ^particles. An effect due to volume/mass can be excluded since the volume of the concentration of 4.5 × 10^11 ^0.05 μm particles approximately corresponds to the volume of the lowest dose of 1 μm (3 × 10^7^) particles, where no effect was observed.

**Table 2:** Summary of stereological results

Particle size/number concentration	APM surface area density [μm^-1^]	Number-weighted mean volume of A549 cells [μm^3^]	Total APM surface area per A549 cell [μm^2^]
Negative control	0.251 (0.043)	1016.3 (92.2)	254.6 (45.9)

1 μm/3 × 10^7^	0.247 (0.036)	1052.2 (69.9)	259.7 (41.8)

1 μm/6 × 10^8^	0.336 (0.048) *	1468.6 (238.4) *	492.5 (98.2) *

0.05 μm/3 × 10^7^	0.247 (0.040)	1090.8 (144.5)	267.3 (43.9)

0.05 μm/6 × 10^8^	0.239 (0.055)	1077.6 (108.9)	256.2 (57.4)

0.05 μm/4.5 × 10^11^	0.376 (0.061) *	1211.6 (75.1)	454.8 (72.2) *

Note. Data are presented as mean (standard deviation). The asterisk (*) indicates a significant difference versus negative control (p < 0.01). The APM surface area density and total surface area per A549 cell were significantly increased at 1 μm/6 × 10^8 ^and 0.05 μm/4.5 × 10^11^. Only at 1 μm/6 × 10^8 ^was the number-weighted mean volume of A549 cells enhanced, probably due to the ingested particles.

### LDH and IL-8 release

**Figure 5:**
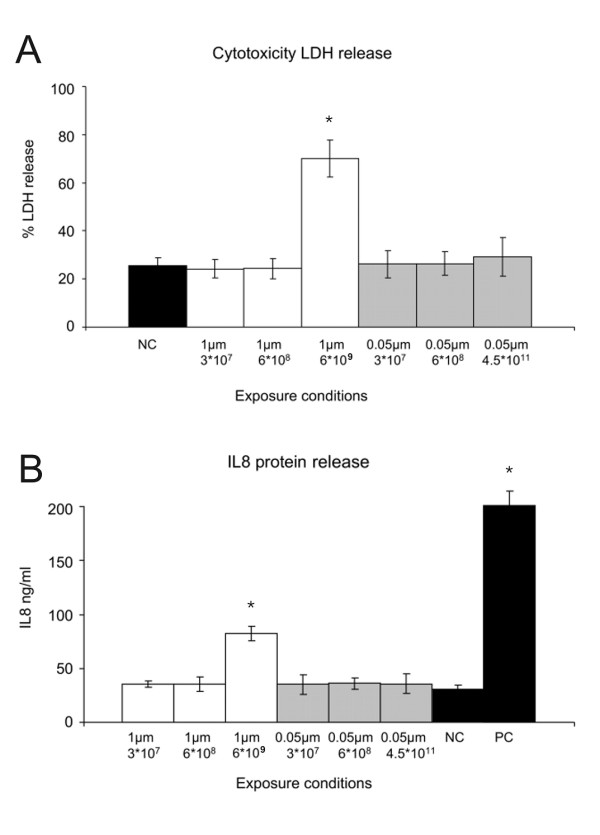
**Cellular LDH release and IL-8 protein after particle exposure**. A: LDH release after 24 h incubation with different concentrations of 1 μm and 0.05 μm particles. An exposure concentration of 6 × 10^9 ^particles per cell culture well significantly increases the LDH release vs. negative control (NC) (* = p < 0.01). B: IL-8 protein after 24 h particle exposure. The concentration of 6 × 10^9 ^1 μm particles induces a significant IL-8 secretion compared to the negative control (NC) (* = p < 0.01). A positive control (PC) was generated with TNFα stimulation. At no other of the tested exposure concentrations was a significant LDH release or IL-8 secretion observed.

A significant increase in LDH and IL-8 release was observed in cells exposed to the highest concentration of 6 × 10^9 ^1 μm particles per cell culture well (Figure 5). Apoptosis and necrosis at this concentration could also be confirmed in transmission electron micrographs. Therefore, this exposure concentration was not included in the APM evaluation. No cytotoxic effects and IL-8 increase were observed at any other exposure concentration of 1 μm and 0.05 μm particles.

### Particle entering into the cells

**Figure 6:**
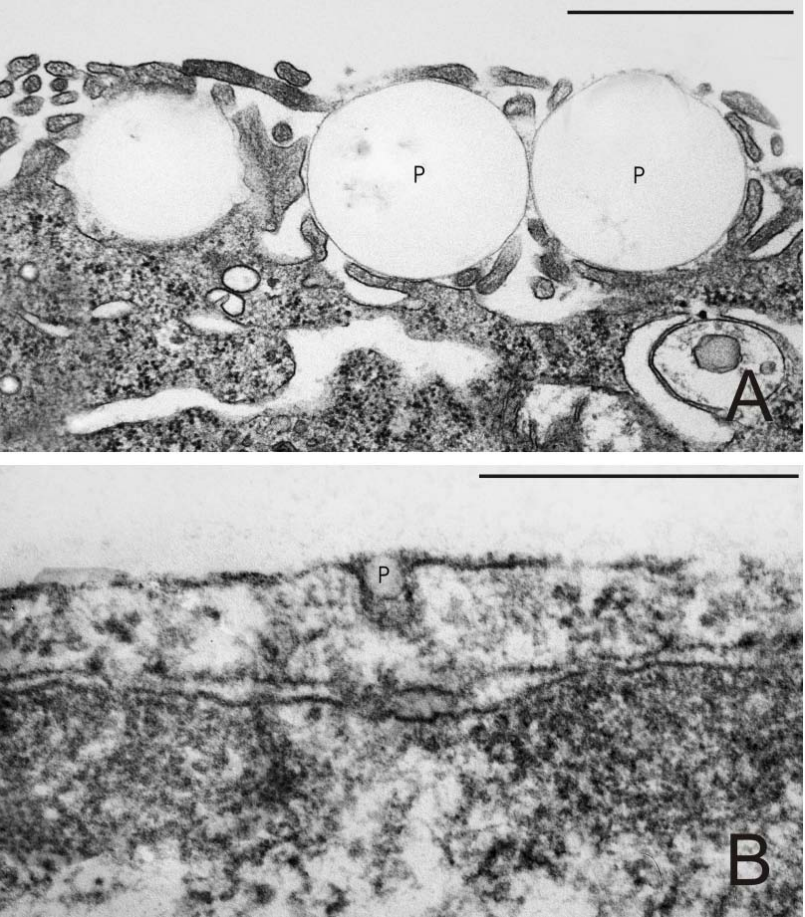
**Electron micrographs of the interaction between particles and the apical plasma membrane of A549 cells**. A: Exposure to 1 μm particles (P). Three particles in the process of cellular uptake, probably via macropinocytosis or phagocytosis. Scale bar = 1 μm. B: Exposure to 0.05 μm particles (P). One particle in the process of cellular uptake, probably via clathrin- or caveolae-mediated endocytosis. Scale bar = 500 nm.

**Figure 7:**
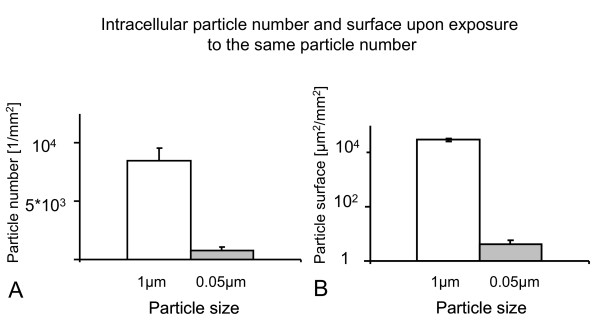
**Intracellular particle number and surface area upon exposure to the same particle number**. Cells were exposed to the same number concentration of particles (3 × 10^7 ^particles per well) and the number of intracellular particles was counted by LSM. From the number of intracellular particles, the total particle surface area taken up by the cells was calculated. There was a greater number (A) and surface (B) of 1 μm particles inside the cells than of 0.05 μm particles. Due to the small sample size (n = 3) and the use of the Mann Whitney u-test, these obvious differences failed to reach statistical significance (p = 0.1). Note the logarithmic scale on the y-axis in B.

**Figure 8:**
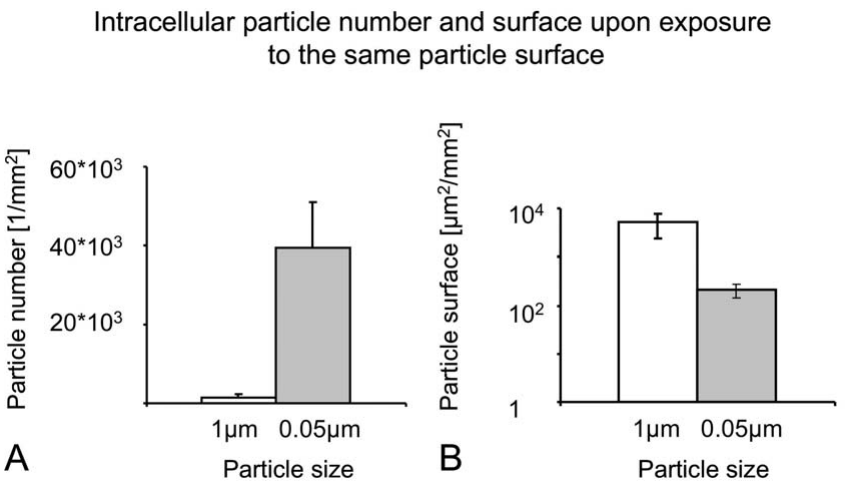
**Intracellular particle number and surface area upon exposure to the same particle surface area**. Cells were exposed to the same total surface area concentration of particles (3.5 × 10^7 ^μm^2 ^per well) and the number of intracellular particles was counted by LSM. From the number of intracellular particles, the total particle surface area taken up by the cells was calculated. At the same surface area concentration, the number of 0.05 μm particles exceeded the number of 1 μm particles taken up by the cells (A), however, the fine particles accounted for a greater intracellular particle surface area (B). Due to the small sample size (n = 3) and the use of the Mann Whitney u-test, these obvious differences failed to reach statistical significance (p = 0.1). Note the logarithmic scale on the y-axis in B.

Qualitatively, differences in cellular uptake were observed between the differently sized particles. The majority of 1 μm particles were taken up by macropinocytosis or phagocytosis (Figure 6A) whereas 0.05 μm particles were rarely observed in the transmission electron microscopic preparations. Figure 6B shows an ultrafine polystyrene particle in the process of uptake by clathrin- or caveolae-mediated endocytosis, identified by morphological criteria. Since particle surface area concentration was shown to be a key parameter of APM increase, A549 cells were either exposed to the same particle number concentration or to the same surface concentration of 1 μm and 0.05 μm particles, respectively. The number of intracellular particles was quantified per mm^2 ^of epithelial cell layer and the intracellular particle surface area was calculated from the intracellular particle number. Figure 7 shows the results of intracellular particle number (A) and particle surface area (B) after exposure to 3 × 10^7 ^1 μm or 0.05 μm particles per well. The number and surface area of intracellular 1 μm particles was higher than that of 0.05 μm particles. After A549 cell exposure to the same total particle surface area concentration (35 mm^2 ^per well), the number of intracellular 1 μm particles was lower than that of 0.05 μm particles but accounted for a higher intracellular particle surface area (Figure 8).

**Figure 9:**
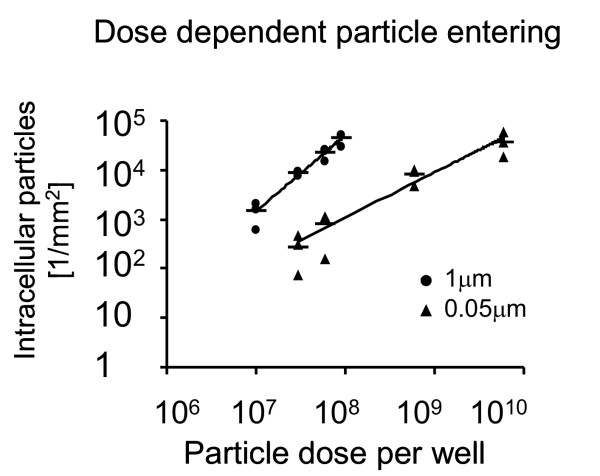
**Dose dependent particle entering**. At rising exposure concentrations, the number of intracellular particles increased for both particle sizes. The increase in the number of 1 μm was steeper than that of 0.05 μm particles. Note the logarithmic scale of the x and y axis. Black circle = 1 μm particles. Black triangle = 0.05 μm particles. Black horizontal line = mean values of three experiments at each concentration. The trend lines are based on the means.

At increasing exposure concentrations, particle entering was quantitatively different between the two particle sizes. Specifically, the number of intracellular 1 μm particles showed a steeper increase than the number of 0.05 μm particles at rising exposure concentrations (Figure 9).

### Transcription of key genes required for lipid synthesis and uptake

Transcription of key genes involved in lipid synthesis and uptake, viz. 3-hydroxy-3-methylglutaryl CoA synthase and reductase (HMG CoA synthase, HMG CoA reductase), fatty acid synthase and low-density lipoprotein receptor (LDL receptor) was analyzed after incubation with 6 × 10^8 ^1 μm particles and 4.5 × 10^11 ^0.05 μm particles for 2 h, 4 h, 8 h, 12 h and 24 h. Only particle concentrations which resulted in an increased APM have been included into the study. The transcription was analyzed by relative expression towards the negative controls. However, no significant increase could be observed at all time points as shown in Table 3.

**Table 3:** Relative expression of genes encoding for proteins involved in lipid synthesis and uptake

Particle size: 1 μm Exposure time	2 h	4 h	8 h	12 h	24 h
HMG CoA Synthase	1.23 (0.34)	1.33 (0.23)	0.80 (0.38)	1.38 (0.42)	1.26 (0.16)
HMG CoA Reductase	1.37 (0.18)	1.12 (0.16)	0.95 (0.16)	1.41 (0.12)	1.33 (0.29)
LDL Receptor	1.33 (0.24)	1.20 (0.21)	1.06 (0.48)	1.24 (0.01)	1.48 (0.31)
Fatty Acid Synthase	1.05 (0.03)	0.91 (0.09)	0.92 (0.34)	0.93 (0.24)	1.26 (0.46)

Particle size: 0.05 μm Exposure time	2 h	4 h	8 h	12 h	24 h

HMG CoA Synthase	1.44 (0.38)	1.11 (0.15)	1.02 (0.12)	1.03 (0.24)	1.00 (0.18)
HMG CoA Reductase	1.08 (0.15)	0.99 (0.17)	0.99 (0.05)	1.03 (0.06)	1.04 (0.29)
LDL Receptor	1.28 (0.20)	1.05 (0.05)	1.06 (0.17)	0.83 (0.11)	1.40 (0.42)
Fatty Acid Synthase	1.15 (0.17)	0.94 (0.04)	1.09 (0.26)	0.97 (0.17)	1.19 (0.40)

Note: Induction of mRNA of each gene relative to the negative control after different exposure times for 6 × 10^8 ^1 μm particles and 4.5 × 10^11 ^0.05 μm particles. The results show the mean of three experiments (standard deviation).

## Discussion

After inhalation, airborne particles are deposited on the surface structures of the respiratory tract. In the alveoli, surfactant displaces particles to the aqueous hypophase bringing them into close contact with the epithelial cells [12,13]. Understanding the interactions between inhaled particles and epithelial cells is of crucial importance because epidemiological and experimental studies have proved that inhalation of airborne particles is associated with adverse effects [5-10]. In recent years, it has been emphasized that particle size differences determine the extent of the cellular reactions to particle exposure and the mechanism by which particles are taken up by epithelial cells [11]. Since the APM of epithelial cells is the first cellular structure the particles encounter, it is particularly necessary to understand whether particles induce changes in the plasma membrane and how they are taken up by the cells. In order to address this issue, we utilized an *in vitro *approach to investigate the effects of particle exposure on the surface area of the APM of A549 epithelial cells. Non-toxic polystyrene particles were used to exclude that cytotoxic or inflammatory effects of the particles influence the observed results. The low toxicity of the particles was confirmed by measuring LDH release and Il-8 secretion (Figure 5). Emphasis was placed on a correlation of dose and effect of different sized particles by analyzing which particle characteristic (number, surface area or mass/volume) showed the strongest correlation between dose and effect. Furthermore, the relation of these changes with the quantitative uptake of particles by epithelial cells was analyzed.

We used A549 cells because it is a widely used and well characterized cell culture line which shares characteristics with alveolar epithelial cells [32,42]. A549 cells are capable of forming various types of endocytotic mechanisms including caveolin- and clathrin-mediated endocytosis [43,44] as well as phagocytosis/macropinocytosis [29]. As the uptake mechanisms of fine and ultrafine particles are very likely to be different from each other [21], the capacity of the cells to perform various endocytic mechanisms is very important. Exposure to a particle suspension was used as it allows an easy and exact dosimetry of particles in terms of particle number. A problem with submersed exposure is that differently sized particles have different diffusion and sedimentation characteristics. Limbach et al. [45] hypothesized that diffusion and sedimentation processes are responsible for a less pronounced uptake of ultrafine particles compared with fine particles. In order to avoid effects of sedimentation, the volume of the particle suspension was kept at a minimal liquid column of 0.8 mm, thus facilitating access of the particles to the cells. To exclude a significant influence of agglomeration of ultrafine particles on our results, we analyzed the size distribution of ultrafine particles in the submersion medium (Figure 1C). Particles were mainly present as single objects or in small aggregates of two to three particles excluding a significant agglomeration.

The analysis of APM surface area changes required the high resolution of the transmission electron microscope in combination with design-based stereology. Stereology allows the unbiased quantification of morphological cellular characteristics in absolute terms, in this case the total apical surface area of the APM per A549 cell. The uptake of particles was suspected to alter total cell volume which was only the case for fine particles at a number concentration of 6 × 10^8 ^particles per well. Since the total APM surface area per cell was calculated from the surface density and the cell volume, this parameter does not depend on changes in the cell volume. The quantification of intracellular particles by confocal LSM and subsequent application of a deconvolution algorithm was shown to be a suitable tool to quantify large numbers of fine and ultrafine fluorescent particles in an efficient way [20]. Nevertheless, it was tested whether only agglomerates of ultrafine particles are recognized or individual ultrafine particles by experiments using 0.05 μm particles emitting fluorescence at different wavelengths. Counting the numbers of particles in the different fluorescence channels separately, and again in the merged channel showed no significant difference between the counts (data not shown), thus verifying the quantification of ultrafine particles.

The main results of our study can be summarized as follows: 1) The surface area of the APM was increased after exposure to 6 × 10^8 ^fine particles and to 4.5 × 10^11 ^ultrafine particles per cell culture well. These results provide evidence for an altered cellular lipid metabolism favoring lipid trafficking to the APM after exposure to high particle concentrations. The increase in APM surface area was dependent on the total particle surface area administered to the cells. The unchanged mRNA expression of key genes required for lipid synthesis and uptake suggests that the additional APM originates from intracellular membrane stores, rather than from new synthesis. 2) At similar number concentrations, the uptake of fine particles was greater than that of ultrafine particles, a relationship becoming more pronounced with increasing particle concentrations. However, upon exposure to equal surface area concentrations, the number of ultrafine intracellular particles exceeded that of fine particles.

The quantification of APM surface area was based on the rationale that the interaction between particles and the membrane might interfere with endocytic and exocytic events leading to an increase or decrease in APM surface area. Interestingly, we observed a significant increase for both particle sizes at equal exposure surface area concentrations, indicating a particle-induced lipid trafficking to the APM. This finding is in accordance with studies on lipid trafficking to the APM, due to deformation stress performed in A549 cells [24]. This study provided evidence that the APM enlargement protects the epithelial cells against injury and helps to reseal plasma membrane injuries. It is reasonable to hypothesize that different mechanic stress stimuli lead to lipid trafficking to the APM to compensate for increases in plasma membrane surface tension [46], loss of membrane after bulk phagocytosis [47] or plasma membrane injury [24,28]. Interestingly, exposure of human embryonic kidney cells and immortalized mouse macrophages to 0.77 μm latex particles caused increased lipid synthesis and induced the expression of genes involved in lipid synthesis and uptake [41]. Lipid synthesis was saturated at a particle concentration of about 30 μg/well of a 96 well plate corresponding to a particle number of 1.29 × 10^8 ^particle per well. Since the wells of the 96 well plate have an area which is approximately 21.4 times smaller than the wells used in the present study, this effect would be expected to occur at a number concentration of 2.5 × 10^9 ^particles or at a surface area concentration of 5 × 10^9 ^μm^2 ^particle surface area. However, in our study the mRNA induction of enzymes involved in lipid synthesis and uptake was not observed at comparably high particle numbers or surface area concentrations. This indicates that the additional membrane in our study stems from pre-existing membrane pools, such as vesicles or the ER [48], rather than from newly synthesized membranes. The lack of the mRNA induction in a non-phagocytic cell line may also explain why the cells die at 1 μm particle number concentrations that are one magnitude higher than those at which the APM increase is observed. The results of this study may not be specific for the respiratory tract epithelium but the comparison with the data of Castoreno et al. [41] shows that the effects of particle endocytosis on lipid metabolism and membrane turnover may depend on the cell type and specialization. It remains to be determined if the lipid synthesis induced by Castoreno et al. [41] and the APM increase analyzed in this study have the same functional origin.

The APM of alveolar epithelial cells serves many functions, including the secretion and re-uptake of surfactant components by type II cells, as well as fluid regulation by type I cells. Any changes occurring in the quantitative composition of the APM of pulmonary epithelial cells may therefore have an effect on the normal metabolism of these cells. We admit that the use of cell lines limits the significance of the observed results for *in vivo *particle exposure in the lung. Not all particles in the alveoli come into contact with the alveolar epithelial cells because alveolar macrophages may take up a major portion of the particles. This makes it difficult to estimate how realistic particle concentrations in a mono cell culture are. However, it has been shown that particularly ultrafine particles interact with the alveolar epithelium [30], partially because they are not taken up by alveolar macrophages as effectively as larger particles [16]. The doses investigated in the present study are higher than usual normal environmental pulmonary exposure, however, tobacco smoking or occupational exposure may increase the number of inhaled particles manifold.

The dependence of APM surface area increase on particle surface area dose corresponds to studies by Stoeger et al. [49,50]. These authors exposed mice to six different particle types and measured the inflammatory response from bronchoalveolar lavage fluid and correlated the effects to the number, surface or mass of the particle dose. They found that particle surface area shows the closest correlation with the inflammatory response [50]. Our present results underline the importance of total particle surface area as the most appropriate dose metric of particles for both structural and functional changes of cells induced by particles.

However, quantification of particles within the epithelial cells shows that the uptake characteristics are different between fine and ultrafine particles. Indeed, extrapolation of the trend lines shown in Figure 9 provides approximately the same numbers of intracellular particles after exposure to 6 × 10^8 ^fine and 4.5 × 10^11 ^ultrafine particles per well, i.e. at the same concentrations that induced the increase in APM surface area. These results offer two reasonable interpretations: (1) Firstly, the exposed surface area determines the number of particles taken up by the cells and the increase in APM surface area independently. (2) Secondly, the main factor influencing increase in APM surface area is not the total particle surface area the cells are exposed to but the number of particles taken up by the cells. These relationships, however, require further analysis before clear conclusions can be drawn.

## Conclusion

In summary, this study demonstrates for the first time that particle exposure induces an increase in APM surface area which correlates with the total particle surface area the cells are exposed to. This increase may be explained by lipid trafficking to the APM and may be interpreted as a protective reaction of the cells against particle induced stress. The uptake of fine particles into the cells was stronger than that of ultrafine particles and this observation gets even more pronounced at increasing particle concentrations. At similar surface concentrations, however, the number of intracellular ultrafine particles may exceed the number of intracellular fine particles.

## Abbreviations

ANOVA: analysis of variance; APM: apical plasma membrane; HMG CoA: 3-hydroxy-3-methylglutaryl Coenzym A; I: intersections; LDH: lactate dehydrogenase; LDL: low-density lipoprotein; LSM: laser scanning microscopy; L_T_: total length of test line; PBS: phosphate buffered saline; S_V_: surface density.

## Competing interests

The authors declare that they have no competing interests.

## Authors' contributions

CB planned the concept and study design, performed the cell culture experiments, LSM analysis, ELISA test, cytotoxicity assay and real-time PCR, interpreted the results and wrote major parts of the manuscript. BRR participated in planning the study design and made substantial contributions to the analysis and interpretation of the data. FB made substantial contributions to the analysis and interpretation of the data. PG contributed to the concept and design of the study and made substantial contribution to the analysis and interpretation of the data. CM planned the concept and study design, performed the stereological and electron microscopic analyses, interpreted the results and wrote major parts of the manuscript. All of the authors have read the manuscript and approved its submission.
